# Early experience with a new integrated microwave surgical device, Acrosurg Revo®, for laparoscopic surgery: A case series of two patients

**DOI:** 10.1016/j.ijscr.2020.12.063

**Published:** 2020-12-24

**Authors:** Yoshitaka Terada, Hiroya Akabori, Hiroyuki Ohta, Yusuke Nishina, Eji Mekata

**Affiliations:** Department of Surgery, National Hospital Organization, Higashi-Ohmi General Medical Center, Gochi-cho 255, Higashiohmi, Shiga, 527-8505, Japan

**Keywords:** Acrosurg Revo®, Laparoscopic surgery, Microwave device, Clean surgical field

## Abstract

**Introduction:**

Abdominal surgery uses various energy devices for vessel sealing, tissue dissection, and detachment. Currently, Acrosurg Revo® (Nikkiso Co., Ltd., Tokyo, Japan), a novel energy device using microwaves, has been developed for use in laparoscopic surgery. This report describes the early clinical experience of using this device in two cases of laparoscopic surgery.

**Presentation of case:**

Case 1 was of a 64-year-old woman who underwent laparoscopic abdominal incisional hernia repair. Case 2 was of a 56-year-old man with a diagnosis of ascending and sigmoid colon cancer who underwent laparoscopic right hemicolectomy and sigmoid colectomy with D3 dissection. Each surgery was completed using Acrosurg Revo® and an endoscopic electrosurgical unit. The postoperative course was uneventful, and both patients were discharged from the hospital without any complications.

**Discussion:**

With this new and novel device, vessel sealing, hemostasis, coagulation, tissue dissection, and detachment were all possible. Notably, there was no spark or mist that hindered the surgical field of view. Furthermore, because microwave coagulation did not result in tissue carbonization, there was a considerable decrease in device tip contamination.

**Conclusion:**

The Acrosurg Revo® may be a useful energy device for laparoscopic surgery.

## Highlights

• Early experience with Acrosurg Revo® in laparoscopic surgery has been described.
• Acrosurg Revo® does not cause sparks or tissue scattering.
• A clean surgical field can be maintained during surgery.
• The Acrosurg Revo®︎ may be a useful energy device for laparoscopic surgery.

## 1 Introduction

**Fig. 1:**
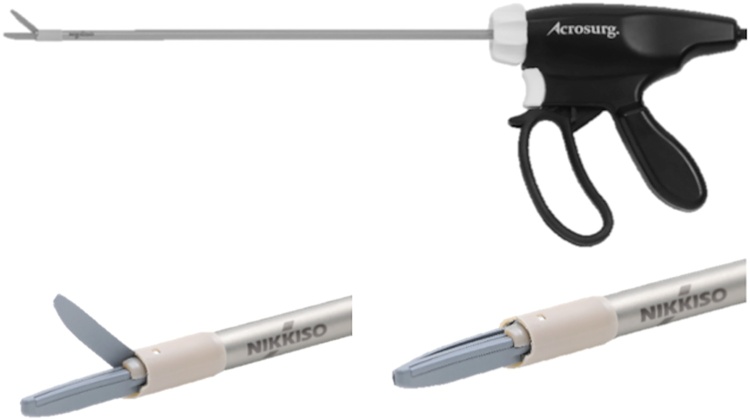
Acrosurg Revo® device. The tip is scissor-shaped, a length that can be used with a laparoscope and rotation of the tip.

Abdominal surgery uses various energy devices for vessel sealing, tissue dissection, and detachment. Previously, Acrosurg® (Nikkiso Co., Ltd., Tokyo, Japan), an energy device using microwaves, was developed as a device for open surgery. Presently, Acrosurg Revo®, a novel device has been developed for use in laparoscopic surgery (Fig. 1) and can be used clinically in japan. This report describes the early clinical experience of using this device in two cases of laparoscopic surgery. Data on these two patients was evaluated retrospectively at our institution. This case series has been reported in line with the PROCESS criteria [1]. This study was registered as a case series on the www.umin.ac.jp website with UMIN000042774.

## 2 Presentation of case

### 2.1 Case 1

A 64-year-old woman with a height of 156 cm, body weight of 79.2 kg, and a body mass index (BMI) of 32.5 kg/m^2^, with a history of laparoscopic cholecystectomy and laparoscopic ovarian tumor resection. She visited our hospital because presented with expansion of the ridge of the umbilical surgical incision over the previous year. On examination, a 40 × 50 mm hernia gate was found on the abdominal umbilical site. Abdominal computed tomography (CT) revealed a fascial defect of 43 × 55 mm above the umbilical site and prolapse of the small intestine. Surgery was performed with a diagnosis of abdominal incisional hernia.

Laparoscopic abdominal incisional hernia repair with intraperitoneal onlay mesh repair and fascial defect closure (IPOM plus) was performed. A 12 mm trocar and two 5 mm trocars were inserted into the left abdomen. When the inside of the abdominal cavity was observed, a fascia defect of 60 × 70 mm was found on the umbilical site, and adhesions of the surrounding abdominal wall and omentum were found. Using Acrosurg Revo®, adhesions of the abdominal wall were removed, and the round ligament of the liver was coagulated and dissected. The fascia defect was sutured closed. The mesh was placed at the exact location, and the edge of the mesh was tacked. The total operation time was 167 min, and blood loss was less than 5 mL. (Video_1,2_SuppInfo)

The patient tolerated food on the day after the surgery and was discharged on day 7 of postoperative care.

### 2.2 Case 2

A 56-year-old man, with a history of diabetes and renal failure and were being treated with insulin, visited our hospital with a complaint of abdominal pain. Lower gastrointestinal endoscopy revealed type 2 advanced cancer in the ascending colon and sigmoid colon (double cancer). Abdominal CT showed a giant mass in the ascending colon, thickening of the sigmoid colon, and swelling of the surrounding lymph nodes. No distant metastasis or peritoneal dissemination was observed. Pathological findings on biopsy showed that both were adenocarcinomas, and the patient was diagnosed with ascending colon cancer with TNM staging (TNM classification 8th) [2] T4aN2bM0, Stage IIIC and sigmoid colon cancer of T2N0M0, Stage I for which surgery was indicated.

Laparoscopic right hemicolectomy and sigmoid colectomy with D3 dissection were performed. Serosa incision was made with a spatula-type laparoscopic electrosurgical unit, and subsequent operations were performed using Acrosurg Revo® alone, without any conventional surgical devices. Specifically, it was used for dissection around the blood vessel, coagulating incisions of small blood vessels including perforating branches, and blood vessel treatment at the time of mesenteric treatment. Vessel dissection was performed at the root of the inferior mesenteric artery and the ileocolic artery, and further D3 dissection was performed. Intestinal anastomosis was performed using double-stapling technique and creating a functional end-to-end anastomosis. The operation time was 516 min, and the blood loss was 400 mL. (Video_3,4_SuppInfo)

The patient tolerated liquids on the day after surgery and food on postoperative day 3. The drain was removed on postoperative day 4, and the patient was discharged on day 15 of postoperative care.

The two patients were being followed up at our hospital after discharge, and had not recurred at the time of reporting.

## 3 Discussion

The two most common energy devices used worldwide are electrothermal bipolar vessel sealing systems (e.g., LigaSure® [Covidien, Minneapolis, USA], and EnSeal® [Ethicon Endo‐Surgery, Cincinnati, USA]), and ultrasonic devices (e.g., Harmonic® [Ethicon Endo-Surgery, Cincinnati, USA], and SonoSurg® [Olympus Medical Systems Corp, Tokyo, Japan]). The bipolar vessel sealing system uses high-frequency electric current and locally irradiates the tissue with a high frequency of 30 kHz-300 MHz to generate Joule heat in the cells to coagulate and seal the tissue. In contrast, ultrasonic devices cause ultrasonic vibration to coagulate and seal tissues by the frictional heat generated between tissue and the device [3,4].

The novel surgical device used in this study, Acrosurg®, uses “microwave” energy. Microwave electromagnetic waves in the 2.45 GHz band act on water molecules in the tissue, and this activated molecular motion heats the tissue itself to cause protein denaturation. The characteristics of microwave energy are as follows: the temperature does not exceed 100 °C, carbonization of the tissue does not occur, and the range of damage around the tissue is minimized owing to the vibration of water molecules. In addition, because it does not spark, there is no generation of mist or scattering of tissue. Tabuse and Katsumi were the first to use a microwave coagulator clinically [5], and Andrew et al. reported ceramic microwaves for ablation of liver tumors [6]. In this device, microwave dielectric heating is generated in the tissue rather than externally, which allows more efficient and uniform transfer of heat throughout the tissue, causing rapid hemostasis during surgery [7]. Recently, we used Acrosurg®, which has vessel sealing, hemostasis, coagulation, tissue dissection, and detachment capabilities [[8], [9], [10]], and reported that this new multifunctional microwave energy device is a safe and useful device for open surgery [11,12]. This time, an improved device for laparoscopic surgery, especially a model with a rotatable tip, was developed as Acrosurg Revo®. This device has several models based on length and can be used in various laparoscopic procedures.

In the first case, Acrosurg Revo® was used for abdominal incisional hernia surgery, and was mainly used for hemostasis at the time of detachment of adhesions and dissection of the round ligament of the liver. Hemostasis, coagulation, dissection, and detachment using the scissors-tip were performed safely without any problems (Video_1,2_SuppInfo). In the second case, it was used for right hemicolectomy and sigmoid colectomy with D3 dissection for the treatment of colon cancer. Fine operations including dissection, incision, and sealing, can be safely performed without any problems (Video_3,4_SuppInfo). A conventional bipolar vessel sealing system heats up with electric discharge and Joule heat, sparks and smoke are often generated. Furthermore, in the ultrasonic device, body fluid or the like is scattered due to friction between the tissue and the device. In laparoscopic surgery, these factors cause obstruction of the operative field of vision. The biggest difference from conventional energy devices is that there was no spark or mist, and a clean operative field can be maintained during surgery. In addition, the number of times the device was replaced was markedly lower because of its multifunctional abilities such as coagulation, hemostasis, and detachment obtained by the microwave energy emitted from the scissor-shaped tip. Finally, contamination at the tip of the device was reduced because the tissue was not carbonized. It is a report in a small number of cases, in the future, it will be necessary to increase the number of cases and compare them with other devices for evaluation.

## 4 Conclusion

Detachment, coagulation, severing, and sealing with the new device were possible without any pitfalls. With less mist and better maintenance of the clean operative field, the Acrosurg Revo® may be a safe-to-use device and can be an alternative to traditional energy devices for laparoscopic surgery.

## Declaration of Competing Interest

The authors report no declarations of interest.

## Funding

No funding or sponsorship.

## Ethical approval

The study is approved by the institutional ethics review board (2-29).

## Consent

Written informed consent was obtained from the patient for publication of this case report and the accompanying images. A copy of the written consent is available for review by the Editor-in-Chief of this journal on request.

The identity of the patients has been protected.

## Author’s contribution

Yoshitaka Terada: Conceptualization, Investigation, Methodology, Data curation, Visualization, Writing - original draft, Writing - review & editing.

Hiroya Akabori: Conceptualization, Methodology, Writing - review & editing, Supervision, Validation.

Hiroyuki Ohta: Data curation, Writing - review & editing, Supervision.

Yusuke Nishina: Visualization, Investigation.

Eji Mekata: Conceptualization, Validation, Writing - review & editing.

Yoshitaka Terada, Hiroyuki Ohta, and Yusuke Nishina have treated surgery and postoperatice care of two patients.

## Registration of research studies

This case report is registered as a case series on the www.umin.ac.jp website with UMIN000042774.

## Guarantor

Yoshitaka Terada.

## Provenance and peer review

Not commissioned, externally peer-reviewed.

## Authorship declaration

In keeping with the latest guidelines of the International Committee of Medical Journal Editors, each author contributed to the case management, follow-up, structuring of the article, and literature review of this case series.

We declare that this manuscript is original, has not been published before, and is not currently being considered for publication elsewhere.

## CRediT authorship contribution statement

**Yoshitaka Terada:** Conceptualization, Investigation, Methodology, Data curation, Visualization, Writing - original draft, Writing - review & editing. **Hiroya Akabori:** Conceptualization, Methodology, Writing - review & editing, Supervision, Validation. **Hiroyuki Ohta:** Data curation, Writing - review & editing, Supervision. **Yusuke Nishina:** Visualization, Investigation. **Eji Mekata:** Conceptualization, Validation, Writing - review & editing.
