# Supplementary material for: Early experience with a new integrated microwave surgical device, Acrosurg Revo®, for laparoscopic surgery: A case series of two patients
Source: Int J Surg Case Rep. 2020 Dec 24;78:375–7. doi: 10.1016/j.ijscr.2020.12.063 (PMC7787920; doi:10.1016/j.ijscr.2020.12.063)
Supplement: Supplementary file 1 [file mmc1.docx]

Video_1_SuppInfo

In Video 1, the small blood vessels of the omentum are coagulated and dissected. There is no problem with vessel sealing and hemostasis.

Video_2_SuppInfo

Video 2, detachment of adhesions. The operability of the tip and the disconnection operation are smooth.

Video_3_SuppInfo

Video 3, round ligament of the liver is coagulated and dissected. Only water vapor is generated, and there is little surgical mist.

Video_4_SuppInfo

Video 4, root dissection of the inferior mesenteric artery could be performed without any problems.
